# Use of the Filovirus Animal Non-Clinical Group (FANG) Ebola virus immuno-assay requires fewer study participants to power a study than the Alpha Diagnostic International assay

**DOI:** 10.1016/j.jviromet.2018.02.018

**Published:** 2018-02-23

**Authors:** James Logue, Kaylie Tuznik, Dean Follmann, Greg Grandits, Jonathan Marchand, Cavan Reilly, Yeya dit Sadio Sarro, James Pettitt, Eric J. Stavale, Mosoka Fallah, Gene G. Olinger, Fatorma K. Bolay, Lisa E. Hensley

**Affiliations:** a Integrated Research Facility at Frederick, National Institute of Allergy and Infectious Diseases, National Institutes of Health, Frederick MD, USA; b University of Minnesota, School of Public Health, Division of Biostatistics, Minneapolis, MN, USA; c Ministry of Health, Monrovia, Liberia; d Division of Clinical Research, National Institute of Allergy and Infectious Diseases, National Institutes of Health, Bethesda, MD, USA; e University Clinical Research Center (UCRC) – SEREFO Laboratory, University of Sciences, Techniques and Technology of Bamako (USTTB), Bamako, Mali

**Keywords:** Ebola virus, ELISA, Serology, FANG, ADI, Antibodies, Immune response

## Abstract

As part of the scientific community’s development of medical countermeasures against Ebola virus disease, optimization of standardized assays for product evaluation is paramount. The recent outbreak heightened awareness to the scarcity of available assays and limited information on performance and reproducibility. To evaluate the immunogenicity of vaccines entering Phase I–III trials and to identify survivors, two enzyme-linked immunosorbent assays, the Filovirus Animal Non-Clinical Group assay and the Alpha Diagnostics International assay, were evaluated for detection of immunoglobulin G against Ebola virus glycoprotein. We found that the Filovirus Animal Nonclinical Group assay produced a wider range of relative antibody concentrations, higher assay precision, larger relative accuracy range, and lower regional background. Additionally, to sufficiently power a vaccine trial, use of the Filovirus Animal Nonclinical Group assay would require one third the number of participants than the Alpha Diagnostics International assay. This reduction in needed study participants will require less money, fewer man hours, and much less time to evaluate vaccine immunogenicity.

## 1. Introduction

Over 11,000 individuals succumbed to the recent and largest Ebola virus disease (EVD) outbreak that occurred in Western Africa (World Health Organization, 2016c; World Health Organization, 2016b). Though many individuals recovered from disease, clear evidence demonstrates that Ebola virus (EBOV) can persist in EVD survivors long after signs and symptoms have subsided (MacDermott and Bausch, 2016; World Health Organization, 2016a). Persistent genomic virus RNA signals have been detected in EVD survivors over a year after resolution of both clinical disease and viremia(Crozier, 2016; Deen et al., 2017; Uyeki et al., 2016). As a result, sexual and vertical transmission of the virus may occur, as evidenced by at least 3 EVD clusters in Liberia, Sierra Leone, and Guinea (Arias et al., 2016; Blackley et al., 2016;Fischer et al., 2016; Fischer and Wohl, 2016; Mate et al., 2015; Sow et al., 2016; Yang et al., 2015). Though the outbreak has ended, countermeasures should be developed to combat any potential reintroductions of EVD into the population by these or other traditional transmission routes. Paramount to these countermeasures is an effective and long-lasting vaccine.

For effective and durable protection, a vaccine must elicit a strong and sustained adaptive immune response against a critical viral target and disrupt the viral replication cycle (e.g., infection, integration, replication). Since the discovery of EVD in 1976, a variety of assays have been developed to detect antibodies targeting filoviruses. While critical in the development of subsequent methods, widespread use of these assays has been limited by reagent quantity, batch-to-batch variability, and a lack of characterization and standardization. These shortfalls are expected as these assays were often developed by researchers for small scale and research-only use; they were not intended for advancement of countermeasures or developed with the intent of seeking regulatory approval. With a standardized assay, the scientific community could compare and replicate assay results across institutions.

Here we present a comparison between two enzyme-linked immunosorbent assays (ELISAs) measuring relative antibody concentrations (rel[Ab]) of immunoglobulin G against EBOV glycoprotein (GP), an immune response thought to be a correlate of protection from death related to EVD (Pushko et al., 2000; Wilson and Hart, 2001; Wong et al., 2012; Xu et al., 1998). The first ELISA was developed by the Filovirus Animal Nonclinical Group (FANG), a consortium of researchers and institutes attempting to standardize multiple aspects of filovirus research. This assay is currently submitted to the Food and Drug Administration for approval as a validated assay. The second ELISA is the commercially available Alpha Diagnostic International (ADI) ELISA. Rel [Ab] range, assay precision, baseline background, correlation between assays, study power requirements, and relative assay accuracy expressed as dilutional linearity were used as points of comparison using identical sample sets for each assay. For this investigation, rel[Ab] range is defined as the range of rel[Ab] observed using each assay for a specific set of samples. Preliminary data collected for the Partnership for Research on Ebola Virus in Liberia (PREVAIL) I vaccine trial suggested that the FANG assay provides less variable rel[Ab] due to plate format, analysis method, and stringent acceptance criteria. Therefore, we applied some aspects of the FANG assay, including a 4-parameter logistic (4-PL) calibrator fit, sample masking criteria, and a pooled serum positive control, to the ADI ELISA to create an optimized ADI assay (ADI^O^). Additionally, analysis methods from the FANG assay were modified and applied to previously collected ADI ELISA data retrospectively (ADI^+^) to increase precision and avoid reprocessing samples using the ADI^O^ assay. As the FANG assay is currently in the process of FDA validation and is not commercially available, we present this ADI^+^ and ADI^O^ assay as alternatives. Due to a lack of reagent and sample availability, the ADI^O^ and ADI^+^ ELISAs were only compared to the FANG and ADI assays to establish assay precision and relative assay accuracy.

## 2. Materials and methods

### 2.1. Origin of samples

Serum samples were collected for a double-blind, randomized, placebo-controlled Phase II efficacy trial of the candidate EBOV vaccines: recombinant chimpanzee adenovirus expressing EBOV GP (rChAd3-EBO-Z) vaccine and recombinant vesicular stomatitis virus modified to replace gene encoding G glycoprotein with the gene encoding EBOV GP (rVSVΔG-ZEBOV-GP) vaccine (Partnership for Research on Ebola Virus in Liberia I trial [PREVAIL I] ClinicalTrials.gov registration no. NCT02344407). Collection of samples from 1500 healthy adult Liberians with no reported history of EVD was approved by the Ministry of Health, Liberia (Kennedy et al., 2016). The protocol used in this clinical vaccine trial was approved by the National Research Ethics Board of Liberia, National Cancer Institute institutional review board, the Liberian Medicines and Health Products Regulatory Authority, and the Food and Drug Administration. Written informed consent was obtained from all the participants.

### 2.2. Experimental design

Adults enrolled in this trial were randomized 1:1:1 to three groups to receive an intramuscular injection of: recombinant chimpanzee adenovirus expressing rChAd3-EBO-Z vaccine (1×10^11^ particle units/ mL; 2 mL), rVSVΔG-ZEBOV-GP vaccine (2×10^7^ particle units/mL; 1 mL), or placebo (saline) (Kennedy et al., 2017). Sera were separated from blood samples collected during the treatment visit (baseline) and 1-month post-vaccination. These samples were processed on the FANG and ADI ELISAs to quantify the antibody response to EBOV GP and to determine the correlation between the two assays.

A positive reference sample was established by equally pooling sera from five EVD survivors enrolled in the PREVAIL III Ebola Natural History Study (NCT02431923) with previously detected high GP-IgG titers as determined by both the FANG and ADI ELISAs. Pooled sera were diluted to form three samples representing high, medium, and low titers (semi-quantitative). EBOV-naïve serum was collected from one healthy, American volunteer. EBOV-naïve sera were also collected from 100 participants in a Mali human immunodeficiency virus study from 2004–2011.

### 2.3. Filovirus animal non-clinical group enzyme-linked immunosorbent assay

The methods used to perform the FANG ELISA were described in the PREVAIL I vaccine trial (Kennedy et al., 2017; Sabourin, 2014). Prior to test sample addition, Immulon 2 HB microplates (Thermo Fisher Scientific, Walkersville, MD, USA) were coated with 100 µL of recombinant EBOV GP and incubated at 4 °C for at least 14 h in the absence of light. Following this incubation, test sample dilutions were prepared in a dilution block in duplicate as a 6-point, 1:2 dilution series on a single plate. The starting dilution of 1:62.5 was prepared using an ELISA diluent containing 5% weight-by-volume dry milk (LabScientific, Highlands, NJ, USA) and 0.1% volume-by-volume Tween-20 (Sigma Aldrich, St. Louis, MO, USA) in phosphate buffered saline (Gibco, Gaithersburg, MD, USA). The reference standard (calibrator) was also prepared in a dilution block as an 11-point, 1:2 dilution series using the ELISA diluent described above. Additionally, a negative control was prepared as a 1:50 dilution using the ELISA diluent, and a high concentration quality control and a low concentration quality control were diluted as a sample processed in singlet. Following this preparation, EBOV GP-coated plates were washed 3 times with 300 µL phosphate buffered saline with 0.1% volume-by-volume Tween-20 (FANG wash buffer) and 100 µL of the sample dilutions, reference standard dilutions, and controls were transferred to the plate. Plates were then incubated at 37 °C for 1 h and washed 3 times with FANG wash buffer, and 100 µL of the horseradish peroxidase-conjugated, anti-human IgG (Thermo Fisher Scientific, Walkersville, MD, USA) was added. Plates were then incubated for an additional hour at 37 °C before washing an additional 3 times with 300 µL of FANG wash buffer and adding TMB Substrate (Thermo Fisher Scientific, Walkersville, MD, USA). Following a 30- minute incubation at room temperature in the absence of light, 100 µL of TMB stop solution (Thermo Fisher Scientific, Walkersville, MD, USA) was added to each well and the plates were read on a SpectraMax Plus 384 plate reader (Molecular Devices, Sunnyvale, CA, USA) at 450 nm. Individual well background optical densities were measured at 650 nm and subtracted from the 450-nm optical density readings prior to performing any calculations. A 4-PL curve were fit to the reference standard using Softmax Pro software provided by the manufacturer. Sample replicate dilutions were masked if optical density readings were outside the bounds of the 4-PL curve. Back-calculated rel[Ab] for each sample replicate dilution were calculated based on the 4-PL calibrator fit and the dilution factor in Softmax Pro. An average and %CV for all sample replicate dilutions were then calculated. If the %CV was greater than a 20% threshold, the sample dilution with a back calculated value furthest from the mean was masked until the %CV fell below that 20% threshold. If the %CV did not fall below 20% or too many dilution points were masked, the sample would fail, and the process was repeated. A sample replicate found to have 0–1, 2–4, or 5 dilution points with optical density readings below the bounds of the reference standard curve required at least 3, 2, or 1 unmasked dilution points to pass acceptance criteria, respectively. If both sample replicates passed, an average rel[Ab] and %CV would be calculated between replicates. If that %CV was greater than 30%, then the sample would fail and be repeated. When all acceptance criteria were met, rel[Ab] were reported.

### 2.4. Alpha Diagnostics International enzyme-linked immunosorbent assay

The ADI ELISA plates were processed according to manufacturer’s instructions (Alpha Diagnostics International, TX, USA). All reagents and plates were provided by the manufacturer. Samples were diluted in dilution blocks as a 6-point, 1:2 dilution series with a 1:250 starting dilution in ADI sample diluent. Then, 100 µL of each sample dilution and ADI plate calibrators, and controls were transferred to ADI microtiter plates pre-coated with recombinant EBOV GP and incubated for 60 min at room temperature. Plates were then washed four times with 300 µL of ADI wash buffer and 100 µL of ADI Anti-Human IgG horseradish peroxidase was added to each well. Following a 30-minute incubation at room temperature, plates were washed an additional 5 times with 300 µL of ADI wash buffer before 100 µL of 3, 3′, 5, 5′-tetramethylbenzidineTMB substrate was added to each well and incubated at room temperature in complete darkness for 15 min. ADI stop solution (100 µL) was then added to each well, and plates were read on a BioTek ELx808 plate reader (Winooski, VT) at 450 nm within 30 min of stop solution addition. Individual well background optical densities were recorded at 630 nm and subtracted from the 450 nm reading prior to any calculations. Rel[Ab] were calculated for each sample dilution based on the line of best fit through the optical densities recorded for the 4 pre-made assay calibrators supplied by the manufacturer using the Gen5 software provided by BioTek. A rel[Ab] of 0 ELISA units per milliliter (EU/mL) was assigned if all dilution points were found to have an optical density below the optical density of the 1 EU/mL calibrator.

### 2.5. Alpha Diagnostics International^+^ enzyme-linked immunosorbent assay

Plates were prepared and processed as described in the ADI method. Unlike the ADI ELISA, rel[Ab] were retrospectively calculated based on a 4-PL model fit (Findlay and Dillard, 2007) to the averaged optical densities for each sample recorded for the 4 pre-made calibrators and the assay blank. Back-calculated rel[Ab] for each average sample dilution were then calculated based on the dilution factor. The %CV threshold of 20% and dilution point masking used for the FANG ELISA were used as acceptance criteria for the ADI^+^ ELISA.

### 2.6. Alpha Diagnostics International^O^ enzyme-linked immunosorbent assay

Serum samples were prepared as described in the ADI method. The 10 unit calibrator was serially diluted 1:2 in ADI sample diluent for a total of six dilution points. Rel[Ab] were calculated for each sample dilution based on the calibrator 4-PL curve of best fit. Additionally, the positive reference (described previously) was processed on every plate as an additional positive control. Back-calculated rel[Ab] for each sample replicate dilution were calculated based on 4-PL calibrator fit and the dilution factor. An average and %CV for all sample replicate dilutions were then calculated. The %CV threshold of 20% and dilution point masking used for the FANG ELISA were used as acceptance criteria for the ADI^O^ ELISA. If both sample replicates passed, an average rel[Ab] and %CV would be calculated between replicates. If that %CV was greater than 30%, then the sample would fail, and the process would be repeated.

### 2.7. Relative antibody concentration range and correlation

The rel[Ab] range following PREVAIL I trial vaccinations were compared using the FANG and ADI ELISAs. The difference in median rel [Ab] between the FANG and ADI assays were compared within each vaccine group at 1-month using the Wilcoxon Signed Rank test. Additionally, Spearman correlations between the rel[Ab] for FANG and ADI assays were calculated for all baseline and 1-month samples by vaccine group to measure agreement between assays.

### 2.8. Vaccine study power comparison

The change in rel[Ab] between baseline and 1-month post-vaccination was compared between the combined active vaccine groups and the placebo group for each assay using Welch’s t-test (non-equal variances). Rel[Ab] were adjusted by adding 1 to every value (to avoid zeros) and log_10_ transforming the data. All rel[Ab] changes are listed in log_10_ EU/mL. The ratio (ADI versus FANG) of sample sizes required to power a future vaccine study using these assays was calculated as the squared ratio of the z-score for each ELISA. A 95% confidence interval for the ratio was calculated using the bootstrap procedure.

### 2.9. Dilutional linearity (relative accuracy)

A high titer, EVD survivor serum pool (positive reference) was serially diluted 1:2 in EBOV-naïve serum to create a total of 9 individual samples encompassing dilutions from 0 to 1:256. Each sample was processed in duplicate on the FANG, ADI, ADI^+^, and ADI^O^ ELISAs by two independent operators. The sample set was then plotted as log_10_ rel [Ab] vs. dilution factor. A linear regression (18 points) model was fit to the data, and a slope and 90% confidence interval were calculated. If the confidence interval was found not to be between 0.7 and −1.2, the most dilute sample was removed until the 90% confidence interval fell within that bound.

### 2.10. Assay precision

Serum from an EVD survivor with a rel[Ab] greater than both seroconversion cutoffs (FANG and ADI cutoffs) described previously was diluted in EBOV-naïve serum to create a total of three samples with a semi-quantitative high, medium, and low GP-IgG rel[Ab] for each assay. Individual sample sets (3) were created for each assay and processed on either the FANG, ADI, ADI^+^, or ADI^O^ ELISA by two distinct operators for a total of 24 replicates for each assay and sample. Assay precision was then established at these three semi-quantitative rel[Ab] by calculating the coefficient of variation.

### 2.11. Regional baseline determination

One hundred presumably EBOV-naïve serum samples from Mali were run on the FANG and ADI ELISAs. A suggested qualitative seroconversion cut-off value for each assay was assigned based on the average rel[Ab] plus three standard deviations using the log_10_ transformed data.

## 3. Results

### 3.1. Relative antibody range and correlation of FANG and ADI ELISAs

**Fig. 1:**
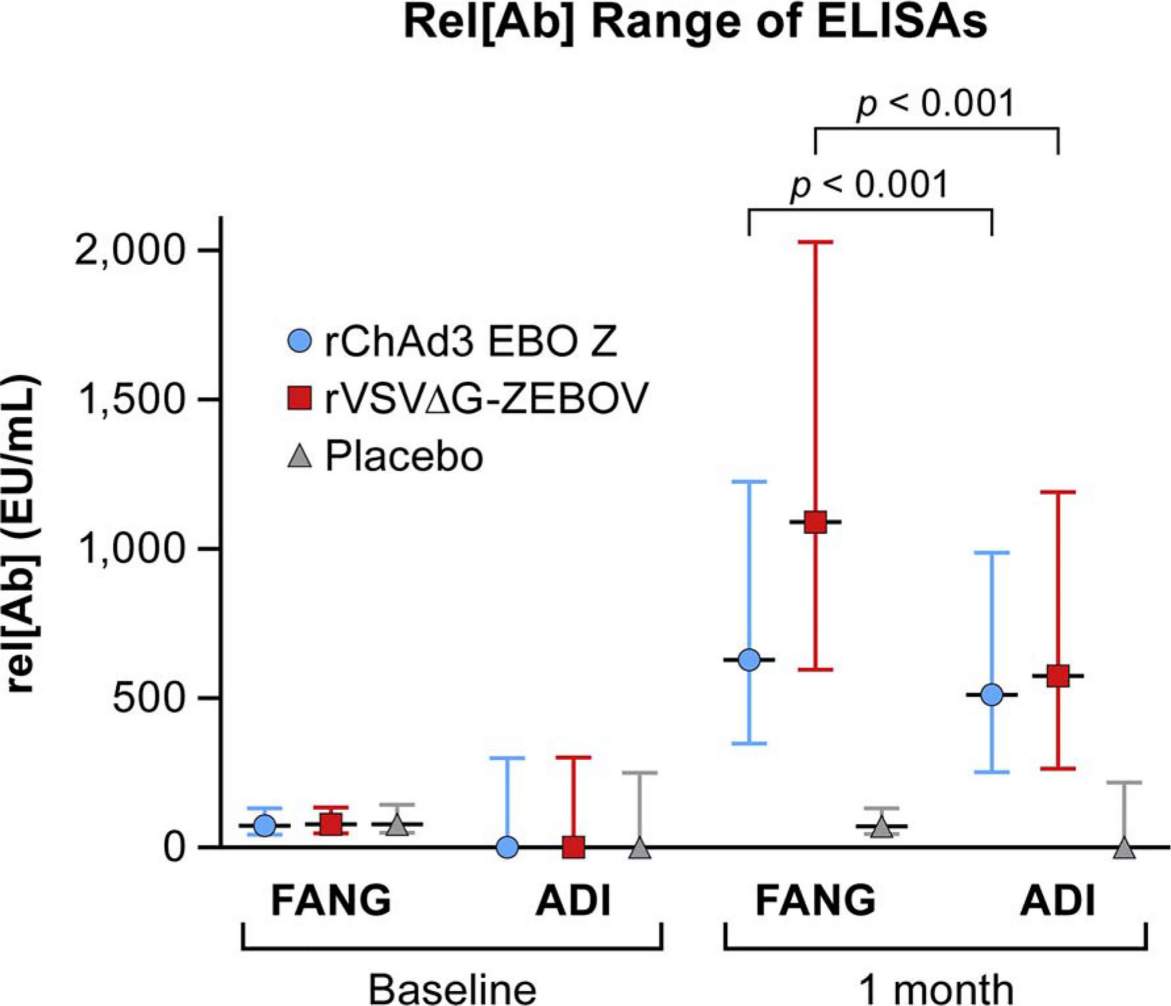
Rel[Ab] Range Comparison of relative antibody concentrations (rel[Ab]) by Alpha Diagnostics International enzyme-linked immunosorbent assay (ADI ELISA) or by Filovirus Animal Non-clinical Group (FANG) ELISA. Median and interquartile range of rel [Ab]s from two vaccine groups, rVSVΔG-ZEBOV-GP (red) and rChAd3-EBO-Z (blue), and placebo (gray).

The median and interquartile ranges (IQR) of rel[Ab] at baseline and 1-month for the 1500 Prevail 1 participants using the FANG and ADI assays are presented in Fig. 1 by treatment group. The median and IQR rel[Ab] humoral vaccine responses for both active vaccine groups observed at 1 month were greater in the samples tested by the FANG ELISA than when these samples were tested by the ADI ELISA (*p* < 0.001 between FANG and ADI for both vaccine groups individually).

Additionally, Spearman correlations between the rel[Ab] for the FANG and ADI ELISAs were calculated to measure agreement between assays. Spearman correlation coefficients ranged from 0.213 and 0.274 at baseline among the three groups and ranged from 0.303 and 0.674 at 1-month (Table 1). Correlations were greater at 1-month for the active vaccine groups than for the placebo group.

### 3.2. Vaccine study power comparison of ADI and FANG ELISAs

**Fig. 2:**
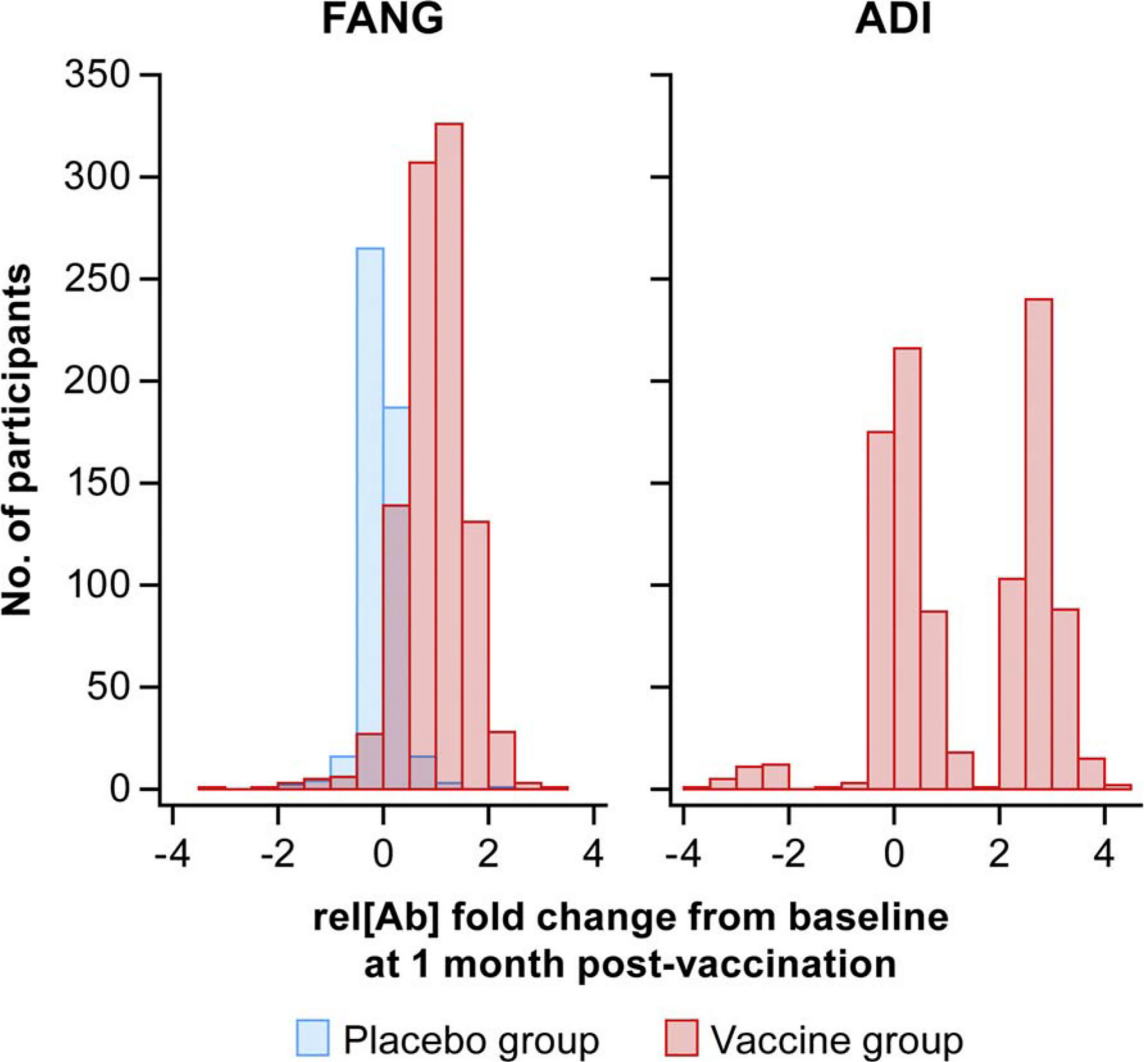
Clinical Trial Vaccine Responses Histogram showing relative antibody concentrations rel[Ab] fold change from baseline in participants at 1-month post-vaccination using the FANG and ADI ELISAs.

The change in rel[Ab] (log scale) for the vaccine and placebo groups as measured on the FANG and ADI ELISAs at 1-month post-vaccination compared to baseline was calculated and the distribution graphed on a histogram (Fig. 2). The average and standard deviation of the changes (log_10_ EU/mL) are given in Table 2. The variation in change within the active groups was smaller with the FANG assay then with the ADI assay, resulting in a larger Z-score when comparing active to placebo groups. The ratio (FANG versus ADI) of sample sizes required to power a vaccine study using these assays was calculated as the squared ratio of the resultant z-scores. Based on the calculated squared z-score ratio of 3.1 (95% confidence interval 2.5, 3.9), we estimate that the ADI ELISA would require about three times as many participants than if the FANG assay was used.

### 3.3. Dilution linearity (relative accuracy) of the ADI, ADI°, ADI^+^, and FANG ELISAs

**Fig. 3:**
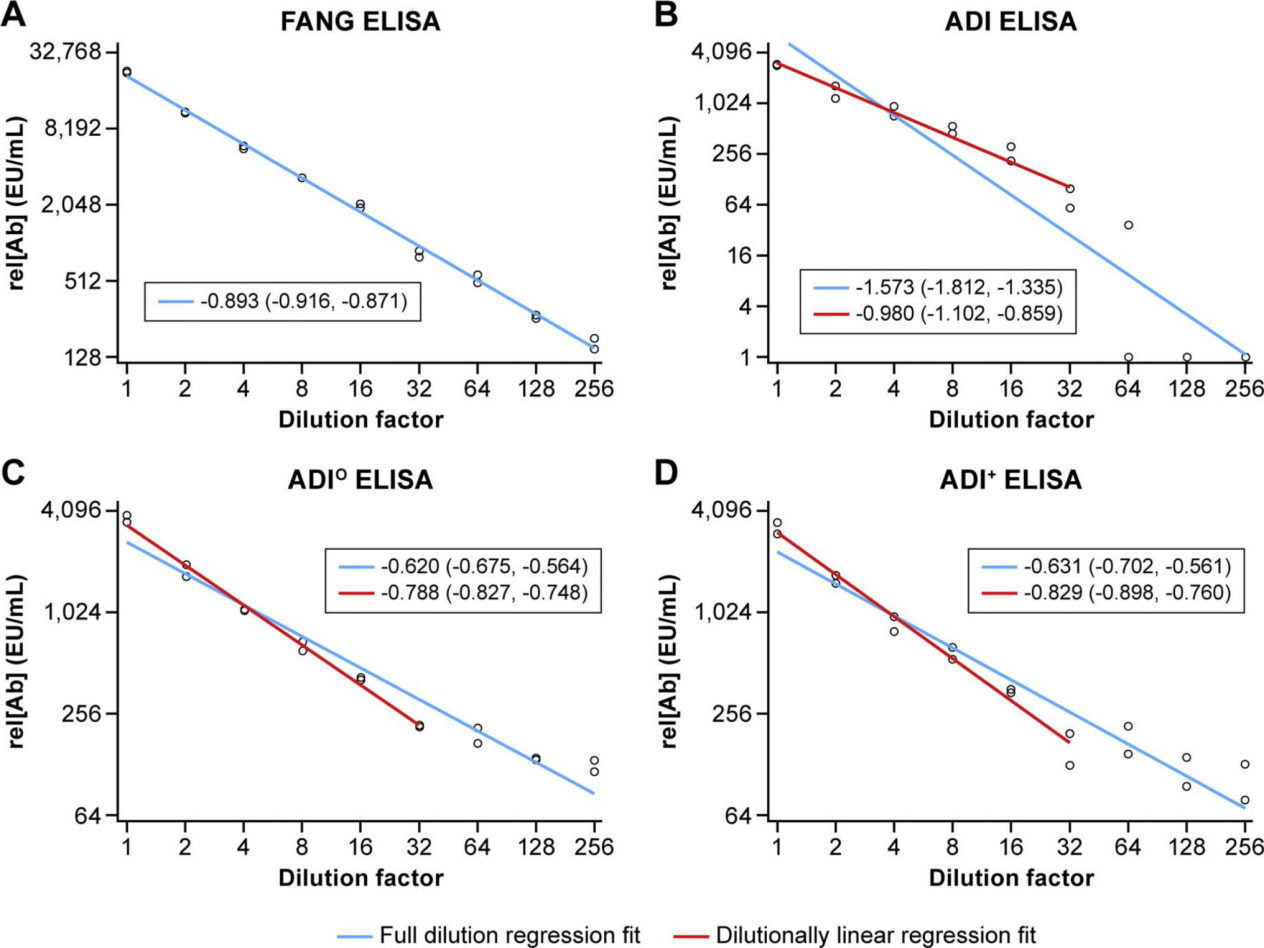
Dilution Linearity Relative antibody concentrations (rel[Ab]) from successive dilutions of serum determined by each enzyme-linked immunosorbent assay (ELISA). The regression line of best fit for each assay are indicated by the solid blue lines. If the original line fit is not dilutionally linear, the dilutionally linear fit is shown in red. Additionally, the slope [90% confidence interval] is listed for each line fit.

Serum pooled from EVD survivors (positive reference) was serially diluted 1:2 in EBOV-naïve serum to create a total of 9 individual samples encompassing dilutions from 0 to 1:256. These samples were processed by the ADI, ADI^+^, ADI^O^, and FANG ELISAs for rel[Ab] and plotted separately for each assay with a regression line of best fit determined (Fig. 3, blue line). If the slope and 90% confidence interval were not found to be between −0.78 and −1.20, the most dilute sample was removed until an acceptable linear fit was found (red line, see methods) The slopes [90% confidence interval] for each full dilution regression fit are: −0.893 [−0.916, −0.871], −1.573 [−1.812, −1.335], −0.620 [−0.675, −0.564], and −0.631 [−0.702, −0.561] for the FANG, ADI, ADI^O^, and ADI^+^ assays, respectively. By the above definition, the full dilution regression fit on the FANG ELISA was found to be dilutionally linear, whereas the ADI assays were not dilutionally linear. The slope [90% confidence interval] for each dilutionally linear regression fit are: −0.980 [−1.102, −0.859], −0.788 [−0.827, −0.748], and −0.829 [−0.898, −0.760] for ADI, ADI^O^, and ADI^+^, respectively. The lower limit of quantification was found to be greater than a 1:256 dilution for the FANG ELISA and a 1:32 dilution for all ADI ELISA methods ().

### 3.4. Assay precision from the ADI, ADI°, ADI^+^, and FANG ELISAs

Coefficients of variation (100×standard deviation / mean; %CV) are shown from repeated measurements (24 replicates per sample) of three samples semi-quantitatively representing low, medium, and high rel[Ab] processed on the ADI, ADI^O^, ADI^+^, and FANG ELISAs (Table 3). Percent CVs were smaller on the FANG assay than for the ADI, ADI^O^, and ADI^+^ assays; this was true for high, medium, and low titer samples.

### 3.5. Regional assay background assessment for the ADI and FANG ELISAs

The average rel[Ab] and standard deviation were calculated for all presumably EBOV-naïve Mali samples (N=100) processed on either the ADI or FANG ELISA (Table 4). A seroconversion cutoff, defined as antibody titers against EBOV larger than 3 standard deviations above the mean (log_10_ transformed), was determined to be 607 and 840 EU/mL (back-transformed), respectively for the FANG and ADI ELISAs. Using these cutoffs, the percentage of participants estimated to seroconvert at 1 month were higher with the FANG assay than with the ADI assay in both vaccine groups (Table 5).

## 4. Discussion

### 4.1. Comparing the FANG and ADI ELISAs

Our extensive processing of samples from the PREVAIL I trial on the FANG and ADI ELISAs has shown both assays to be acceptable ELISA platforms. Both assays showed large and significant changes in rel[Ab] in participants that were vaccinated with active vaccine. These assays can be used in a variety of laboratory spaces and locations, including field locations. While the correlations shown in section 3.1 highlight that both assays trend similarly when measuring patient serum samples for IgG against EBOV GP, the FANG assay had some notable advantages.

First, as made evident by the PREVAIL I vaccine data set, the rel[Ab] range of the FANG ELISA exceeds the range of the ADI assay, allowing an investigator to better compare vaccine responses. Though not tested in this study, rel[Ab] range differences could be due to a number of factors, including antigen concentrations on the plate, secondary antibody concentrations, or differences introduced by antigen manufacturing or coating processes. However, as the ADI ELISA is proprietary, differences between antigen manufacturing and plate coating processes cannot be properly compared. Additionally, the different instruments used to obtain optical density readings for the FANG and ADI ELISAs in this work could have an effect on the ranges reported here. Yet, as both instruments were routinely maintained, any potential differences in values would most likely be negligible. Interestingly, two distinct groups of PREVAIL I vaccine responders were observed using the ADI ELISA as opposed to the single bell-curve distribution observed for the FANG ELISA (Fig. 2). This bimodal distribution seems to suggest that the ADI ELISA was not able to detect the full response to the vaccine in all participants. As similar proportions of individuals receiving each of the two vaccines appear to seroconvert using the ADI ELISA (Table 5), it is unlikely that the vaccine type contributed to this effect. However, further study will be needed to fully investigate why this distribution is observed using the ADI ELISA.

Second, the FANG ELISA exceeds the ability of the ADI assay to accurately and precisely determine the rel[Ab] of diluted samples as expressed by dilutional linearity and assay precision. Due to sample availability, a serum pool from EBOV survivors as opposed to vaccinated individuals was used to assess the precision and relative accuracy of these two assays. Another important distinction between assays is the level of background rel[Ab] associated with a presumably naïve population (100 naïve persons from Mali). In this group, the standard deviation and average rel[Ab] were smaller on the FANG ELISA than the ADI ELISA. Thus, a higher seroconversion cutoff is associated with the ADI assay, and discerning whether an individual seroconverts is hampered as compared to the FANG ELISA, as evidenced by the lower percentage of individuals who appeared to seroconvert in the PREVAIL I clinical vaccine trial (Table 5).

Most importantly, however, based on the PREVAIL I vaccine responses and the z-scores comparing active vaccine groups to placebo groups (Table 2), we estimate that the use of the ADI ELISA would require about three times as many participants to power a future vaccine study than if the FANG assay was used. Due to the reduction in needed study participants associated with the FANG assay, vaccine efficacy will be determined more quickly and will likely require less labor and cost. For these reasons, though both assays are shown to be versatile for use in multiple locations, we would suggest using the FANG assay to quantify rel[Ab] of IgG against the EBOV GP. Though this work does not necessarily translate to immune response quantification in other species, we suspect similar advantages will be seen using the FANG ELISA in animal models of EBOV infections. However, work comparing these ELISAs in non-human primate and other animal research has yet to be completed.

### 4.2. ADI^O^ and ADI^+^ ELISA comparison

As the FANG assay is not yet widely available, optimization methods were developed for the commercially available ADI assay for use in the field. Specifically, a 4-PL calibrator curve fit (Findlay and Dillard, 2007) and stringent acceptance criteria were applied to both the ADI^O^ and ADI^+^ optimization methods. A positive serum control was also added to the ADI^O^ ELISA. As mentioned previously, assay precision and relative accuracy were the only aspects of these optimization methods assessed due to reagent and sample availability restrictions. Therefore, conclusions regarding rel[Ab] range and study participant requirements using the optimization methods could not be properly assessed through this work. Though no improvements in relative accuracy were observed between the ADI assay and the ADI^+^ and ADI^O^ methods, precision increased for both optimization methods at the low and medium titer samples. Given these data and that the FANG assay is not yet commercially available, the ADI assay can be a good alternative when necessary, especially when the optimization and reanalysis steps (i.e., ADI^O^ and ADI^+^) described above are applied. The comparison presented demonstrates the importance of certain aspects of ELISA platforms in generating a more precise result, including a 4-PL curve fit, stringent acceptance criteria and the use of well qualified matrix controls.

## Figures and Tables

**Table 1:** Spearman correlation between rel[Ab] obtained from the ADI and FANG ELISAs by evaluation time.

Time of evaluation	rChAd3 EBO Z	rVSVΔG-ZEBOV GP	Placebo
Baseline	0.213	0.269	0.274
1 mo post-vaccination	0.584	0.674	0.303
Total no. tested	487	484	491

ChAd3 EBO Z, chimpanzee adenovirus type 3 expressing Ebola virus glycoprotein Yambuku variant; rVSVΔG-ZEBOV, recombinant vesicular stomatitis virus with substitution of gene for Ebola virus glycoprotein for the VSV G glycoprotein.

**Table 2:** The change in rel[Ab] on each ELISA from baseline to 1-mo post-vaccination by treatment groups.

ELISA type	Avg rel[Ab] difference log_10_ EU/ml (SD), Vaccine groups	Avg rel[Ab] difference log_10_ EU/ml (SD), Placebo group	Z score
FANG	0.964 (0.613)	−0.043 (0.326)	41.15
ADI	1.314 (1.469)	−0.221 (1.024)	23.33
Total tested	978	494	

ADI, Alpha Diagnostic International; ELISA, enzyme-linked immunosorbent assay; FANG, Filovirus Animal Nonclinical Group; rel[Ab], relative antibody concentration.

**Table 3:** %CV of rel[Ab] at increasing arbitrary titers processed repeatedly on different types of ELISAs.

Sample titer	FANG ELISA CV %	ADI ELISA CV %	ADI^O^ ELISA Optimized CV%	Retrospective ADI^+^ ELISA data reanalyzed CV%
High titer	15.8	23.3	21.8	23.9
Medium titer	13.5	21.9	15.0	17.5
Low titer	8.8	71.1	40.8	36.0

ADI^O^, optimized ADI ELISA, ADI^+^, reanalysis of data obtained from commercially available ADI ELISA, CV, coefficient of variation; ELISA, enzyme-linked immunosorbent assay; FANG, Filovirus Animal Nonclinical Group; rel[Ab], relative antibody concentration. N=24 replicates for each sample.

**Table 4:** Average background rel[Ab] against EBOV glycoprotein in EBOV-naïve Mali participants detected by different ELISAs.

ELISA type	Avg rel[Ab] (EU/ml)	SD of rel[Ab] (EU/ml)	Seroconversion cutoff^a^ (EU/ ml)
FANG	104.1	156.9	607
ADI	199.4	224.3	840

a Seroconversion cutoff is defined as antibody titers against EBOV of 3 standard deviations above the mean (log_10_ transformed).

ADI, Alpha Diagnostic International; EBOV, Ebola virus; ELISA, enzyme-linked immunosorbent assay; FANG, Filovirus Animal Nonclinical Group; rel[Ab], relative antibody concentration; SD, standard deviation.

**Table 5:** Percentage of vaccine participants who seroconverted by 1 month following vaccination determined by different ELISAs.

ELISA type	rChAd3 EBO Z responders (%) N=487	rVSVΔG-ZEBOV responders (%) N=484	Placebo N=491
FANG	250 (51.3%)	358 (74.0%)	19 (3.9%)
ADI	150 (30.8)	167 (34.5%)	29 (5.9%)

ELISA, enzyme-linked immunosorbent assay, FANG, Filovirus Animal Nonclinical Group. Seroconversion cutoffs for the FANG and ADI ELISAs were 607 and 840 EU/ml, respectively.
